# Supplementary material for: Identification of photoperiod-induced specific miRNAs in the adrenal glands of Sunite sheep (Ovis aries)
Source: Front Vet Sci. 2022 Jul 22;9:888207. doi: 10.3389/fvets.2022.888207 (PMC9354845; doi:10.3389/fvets.2022.888207)
Supplement: Supplementary file 1 [file Data_Sheet_1.ZIP › Supplementary file/miRDeep2 file.zip!/miRDeep2 file/Novel_Structure/SPLP42A3/Novel_44.pdf]

Provisional ID	:	NC_019459.2_4183
Score total	:	1.9
Score for star read(s)	:	-1.3
Score for read counts	:	0
Score for mfe	:	1.6
Score for randfold	:	1.6
Score for cons. seed	:	
Total read count	:	30
Mature read count	:	28
Loop read count	:	0
Star read count	:	2

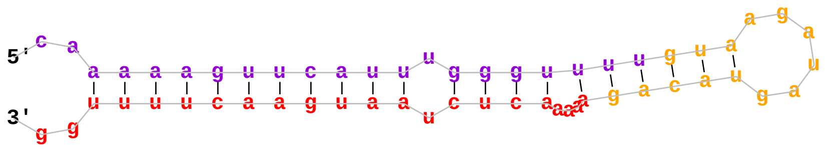

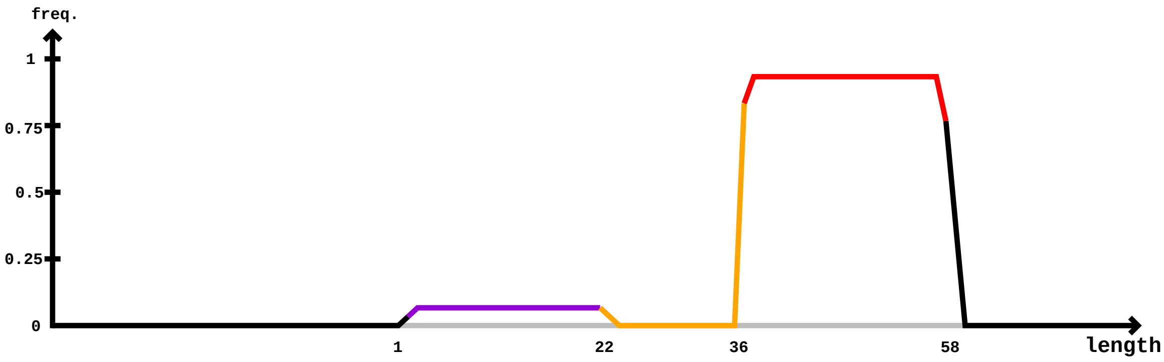


```
                                   Star                             Mature
5'- gggcugaauaauauuccacuauauaguggguuggccaaaaaguucauuuggguuuuguaagauaguacagaaaaacucuaaugaacuuuuggggcaacccaauguauuuau   -3'    obs
    gggcugaauaauauuccacuauauaguggguuggccaaaaaguucauuuggguuuuguaagauaguacagaaaaacucuaaugaacuuuuggggcaacccaauguauuuau          exp
    (((...........)))...(((((.(((((((.((.(((((((((((.((((((((((......))))))...)))).))))))))))).)).))))))).))))).... reads   mm   sample
    ...................................caaaaaguucauuugCguuuu.......................................................  1       1    seq
    ....................................aaaaaguucGuuuggguuuug......................................................  1       1    seq
    ......................................................................aaaaacucuaaugaacuuuug....................  2       0    seq
    ......................................................................aaaaacucAaaugaacuuuugg...................  23      1    seq
    .......................................................................aaaacucAaaugaacuuuug....................  3       1    seq
```
